# Too cold or too warm? Modelling seed set and fruit mass based on the effect of temperature on pollen quality

**DOI:** 10.1093/aobpla/plag004

**Published:** 2026-02-02

**Authors:** Ana Cristina Zepeda, Stefan Vorage, Simon van Mourik, Ep Heuvelink, Leo F M Marcelis

**Affiliations:** Horticulture and Product Physiology, Department of Plant Sciences, Wageningen University, Droevendaalsesteeg 1, 6708 PB Wageningen, The Netherlands; Horticulture and Product Physiology, Department of Plant Sciences, Wageningen University, Droevendaalsesteeg 1, 6708 PB Wageningen, The Netherlands; Agricultural Biosystems Engineering, Department of Plant Sciences, Wageningen University, Droevendaalsesteeg 1, 6708 PB Wageningen, The Netherlands; Horticulture and Product Physiology, Department of Plant Sciences, Wageningen University, Droevendaalsesteeg 1, 6708 PB Wageningen, The Netherlands; Horticulture and Product Physiology, Department of Plant Sciences, Wageningen University, Droevendaalsesteeg 1, 6708 PB Wageningen, The Netherlands

**Keywords:** pollen viability, pollen germination, seed set, fruit set, reproductive processes, modelling fruit mass

## Abstract

Seed and fruit set are processes determining yield in many crops. However, many growth models for horticultural crops do not explicitly incorporate these processes. The aim of this study was to develop a quantitative model to predict seed set, fruit set, and fruit mass based on the effects of temperature and duration of a period with high or low temperature on pollen number and pollen quality (viability and germination fraction). To develop the model, we conducted an experiment where fruiting dwarf tomato plants were grown at 18°C and exposed to 14°C for 4, 6, or 8 days, 30°C and 34°C for 1, 3, or 4 days, and a control treatment at 18°C continuously. Temperatures of 30°C and 34°C reduced pollen viability and germination, resulting in lower seed set and fruit mass. While fruit set remained unaffected at 30°C, both 14°C and 34°C led to reduced fruit set. At lower temperatures (14°C), our model predicted decreased yields due to a lower number of fruits in the truss, resulting from reduced fruit set and smaller fruit size compared to the reference temperature (18°C). At higher temperatures (30°C), our model predicted reduced fruit yield due to smaller individual fruit size, resulting from low seed set. Our research introduces a modelling framework that accounts for the influence of periods with high or low temperature on seed set, a process that is almost never considered in growth models for horticultural crops. This framework is crucial for developing strategies to optimize crop yield in response to temperature fluctuations.

## Teaser

Seed and fruit set are processes determining yield in many crops. However, many growth models for horticultural crops do not explicitly incorporate these processes. We developed a quantitative model to predict seed set, fruit set, and fruit mass based on the effects of temperature and duration of a period with high or low temperature on pollen number and pollen quality (viability and germination fraction). By explicitly capturing these mechanisms, the model improves yield prediction under fluctuating temperatures.

## Introduction

Seed and fruit set are determined shortly after fertilization and define the number and the final mass of fruits, thereby establishing yield potential (Ruan et al. 2012). These reproductive processes are highly sensitive to environmental stress, particularly temperature extremes, which can lead to seed and fruit abortion (Zinn et al. 2010, Hedhly 2011, Ruan et al. 2012). Although fruit and seed set are key components to predict yield, and extreme temperatures are becoming more common with climate change (Pereira et al. 2022), reproductive processes remain simplified in most horticultural crop models (Marcelis et al. 1998, Poorter et al. 2013). This simplification is largely due to limited quantitative physiological data and the difficulty of parameterizing mechanistic models describing reproductive processes (Boote et al. 2013). However, without explicitly modelling these processes, crop models risk large errors in predicting fruit mass and yield, limiting their usefulness in reliably estimating yield losses (especially under fluctuating climate) and in effectively guiding climate-control strategies under variable conditions.

Fruit set refers to the transition from an ovary to a growing young fruit that occurs after pollination and fertilization (Ruan et al. 2012) or, in the case of parthenocarpy, after hormonal signals (Pandolfini et al. 2009). Seed set is the process by which fertilized ovules develop into seeds, a process that strictly requires fertilization (Ruan et al. 2012). Successful fruit and seed production relies on the successful outcome of sequential processes such as pollination, pollen tube growth, and fertilization of ovules. These processes depend on the formation of viable pollen grains, which is highly sensitive to temperatures (Santiago and Sharkey 2019). Therefore, pollen development is a key step linking non-optimal temperatures to reduced yield. In tomato, pollen development is particularly sensitive to high temperatures about 8–15 days before anthesis (Sato et al. 2002). High temperatures during this period can reduce pollen production by up to 40% (Prasad et al. 1999, Sato et al. 2000, Pham et al. 2020) and impair viability and germination, limiting the formation of seeds (Chaturvedi et al. 2021). Seeds have been suggested to act as both sources and sinks for cytokinins and auxins, thereby stimulating cell division and expansion in developing ovaries (Bohner and Bangerth 1988), which can explain why fruit size is often positively correlated with seed number (Picken 1984, Marcelis and Baan Hofman-Eijer 1997, Peet et al. 1998, Okello et al. 2012). Nevertheless, seed set is rarely included in crop models.

Surprisingly, progress in simulating reproductive processes in growth models for horticultural crops has been limited, despite the well documented impact of high non-optimal temperatures on pollen, fruit, and seed development. This is particularly evident in tomato growth models, where fruit set is often included as a fixed input (Heuvelink 1996), or, at best, modelled as a linear function of assimilate availability relative to the number of sinks (Vanthoor et al. 2011). This simplified approach is problematic because high-value crops in controlled environments can lose substantial yield from even brief high- or low-temperature episodes (Santiago and Sharkey 2019). More progress has been made for open-field crop growth models, where temperature effects on yield are incorporated by simulating pollen germination probabilities (Coast et al. 2016), percentage of seed set (Singh et al. 2017), or spikelet sterility (Julia and Dingkuhn 2013). However, these approaches ultimately link a single process to reduced fruit yield. Without accounting for the temperature sensitivity of each reproductive stage, models cannot reliably predict losses or effectively guide climate-control strategies in variable climate conditions.

The aim of this study is to develop a quantitative model for tomato that predicts the temperature response of seed set and fruit mass. The model represents reproductive success as the outcome of sequential processes (pollen formation, pollen viability, pollen germination, and seed set), each influenced by temperature at varying exposure levels. To develop the model, we conducted an experiment with dwarf tomato plants at different developmental stages, grown at 18°C and exposed to 14°C for 4, 6, or 8 days, 30°C and 34°C for 1, 3, or 4 days, and a control treatment at 18°C continuously.

## Materials and methods

### Plant material and growth conditions

Tomato (*Solanum lycopersicum* cultivar F1 2414, Vreugdenhil, the Netherlands) seeds were sown in trays with potting soil mix covered with a thin layer of vermiculite and stored in a dark cold room at 4°C. After 24 h, the trays with the seeds were moved to a climate cabinet (Weiss Technik, the Netherlands), where temperature was set at 18°C, relative humidity 70% and light intensity 230 µmol m^−2^ s^−1^ during a 16-h photoperiod, at ambient CO_2_. Light was provided by white LED modules (Hettich Benelux B.V.). The desired photosynthetic photon flux density at canopy level was maintained by dimming the lamps. Seedlings were transplanted 25 days after sowing (DAS) to 11 × 11 × 12 cm plastic pots filled with the same potting soil and distributed over three climate cabinets with the same climate conditions as mentioned above. The cultivation area of each climate cabinet was 0.84 m^2^ and plants were grown at 30 plants m^−2^ Plants were watered with a nutrient solution (electrical conductivity 2.1 dS m^−1^, pH 5.5) containing 1.2 mM NH_4_^+^, 7.2 mM K^+^, 4.0 mM Ca^2+^, 1.8 mM Mg^2+^, 12.4 mM NO_3_^−^, 3.3 mM SO_4_^2^, 1.0 mM PO_4_^2−^, 35 μM Fe^3+^, 8.0 μM Mn^2+^, 5.0 μM Zn^2+^, 20 μM B, 0.5 μM Cu^2+^, 0.5 μM MoO_4_^2−^. Only the first three trusses on the main stem were kept, and all additional trusses were removed as soon as they were visible. The three trusses were numbered according to their order of appearance. All trusses were pruned to 9 flower buds per truss to eliminate potential treatment effects on flower initiation. We removed all axillary shoots and additional trusses, leaving only three trusses with nine flowers each across all treatments. Once the first flower was open, we gently shook the plant's stem every other morning to facilitate self-pollination of the flowers. In addition, throughout the experiment, emerging lateral shoots were removed weekly.

### Treatments and experimental set up

We conducted an experiment with two factors (temperature and duration of the temperature) and a control treatment, resulting in a total of 10 treatments. The control treatment was maintained at constant 18°C (day and night), considered the optimal growth temperature. For the other treatments, the temperature was reduced to 14°C during 4, 6, or 8 days or increased to 30°C and 34°C during 1, 3, or 4 days. Different durations were used for the different temperatures, because low temperatures require several days to affect reproduction, whereas high temperatures can cause damage within hours to a few days (Ohnishi et al. 2010, Chaturvedi et al. 2021). Longer durations for low temperatures were needed to capture the threshold at which effects appear.

Temperatures were constant day and night. Temperature treatments were applied just before the first flower of the first truss reached anthesis (approximately 49 days after sowing). At this moment, the second and third trusses were already present but were less developed. The same vapour pressure deficit was maintained at 0.65 kPa in all treatments, resulting in a relative humidity of 88%, 85%, 61%, and 70% at 34°C, 30°C, 14°C, and 18°C, respectively. The experiment was carried out using five available climate cabinets. Three cabinets were set to 14°C, 30°C, or 34°C, while the remaining two cabinets were set to 18°C. The plants were moved between cabinets according to the duration of the temperature stress. After all treatments were completed, the temperature and humidity were reset to the standard conditions of 18°C and 70% relative humidity in all cabinets.

### Pollen number and viability

To determine the pollen number per flower and pollen viability fraction, the first flower (position 1) of each truss (1, 2, or 3) was collected once it was fully open. The anther cone was removed from the flower, inserted into a 1.5 ml Eppendorf tube. Tubes were added with 1.5 ml Carnoy’s fixation solution (6 Ethanol: 3 chloroform: 1 glacial acetic acid, as described in Kearns and Inouye 1993) to enable storage of the cone until measuring time. At measuring time, the anther cone was dried on tissue paper, dissected into four pieces with a sharp razor blade, and inserted again inside a 1.5 ml Eppendorf tube. Pollen was stained by adding 200 µl of Alexander’s dye (Alexander 1969) to the Eppendorf tube, vortexed for 20 s, and stored at room temperature for at least 24 h before observation. Alexander’s dye consisted of 10 ml of 95% ethanol, 1 ml of malachite green (1% solution in 95% ethanol), 54.5 ml of distilled water, 25 ml of glycerol, 5 ml of acid fuchsin (1% solution in water), 0.5 ml of orange G (1% solution in water) and 4 ml of glacial acetic acid for a 100 ml solution (Kearns and Inouye 1993). After 24 h of staining, 10 µl of pollen suspension was loaded into a Fuchs–Rosenthal haemocytometer (4 × 4 × 0.2 mm grid, 3.2 mm^3^). The counting chamber was observed under a LEICA MZ APO stereomicroscope equipped with an Axiocam 305 color camera controlled by Axio Vision 4.8.1.0 software. The counting chamber was divided into 16 large cubes (1 × 1 mm, 1 mm^2^), and each cube was further divided into 16 small squares (0.25 × 0.25 mm, 0.0625 mm^2^). Two large squares (1 mm^2^ each) were randomly selected per load, and pictures were made to further count viable and unviable pollen with a cell counter plugin of Fiji—ImageJ (version 1.53c). For each sample (flower), the haemocytometer was loaded two times; therefore, the average viable, unviable, and total pollen number per flower was based on a total of four pictures (Supplementary Figure S1). Pollen grains that appeared dark purple after staining were classified as viable, and those that remained transparent were classified as dead (unviable). The fraction of viable pollen was calculated as the ratio of viable to total (viable and unviable) pollen grains on a 10 µl aliquot.

The total number of pollen per flower ($N_{\;pollen,mes}$, #pollen per flower) was calculated as


(1) $$\begin{array}{c} N_{\;pollen,mes}=\frac{N_{\;pollen,count}\;}{vol_{chamber}}\cdot vol_{sample}, \end{array}$$

where $N_{\;pollen,count}$ is the number of pollen counted (#), $vol_{chamber}$ is the haemocytometer chamber volume (0.2 mm^3^), and $vol_{sample}$ indicates the sample volume (200 µl), which corresponds to the initial volume in which the flower was suspended.

### Pollen germination

To determine pollen germination fraction, the second flower (position 2) of each truss (1, 2, or 3) was collected once it was fully open. Daily observations of the chamber were made to record the exact time of flower opening. We harvested flowers from four different plants in each repetition for these measurements. The anther cone was immediately dissected into four pieces inside a 1.5 ml Eppendorf tube where 200 µl of germination solution was added and vortexed for 20 s to suspend the pollen in the solution. The germination solution consisted of 100 g L^−1^ sucrose, 2 Mm boric acid, 2 Mm calcium nitrate, 2 Mm magnesium sulphate, and 1 Mm potassium nitrate (Firon et al. 2006). The pollen suspension was placed in a petri dish (3.5 cm diameter) with 1 ml agar layer previously solidified (8 g of agar per L germination solution as previously described for the suspension). The agar with the pollen suspension was left for 1 h to germinate at room temperature. Germination was observed with a Leica MZ APO stereomicroscope equipped with an Axiocam 305 colour camera controlled by Axio Vision 4.8.1.0 software. Two random areas, each measuring 0.9 mm^2^, were selected, and a picture of each area was taken to further count pollen germination with a cell counter plugin of Fiji—ImageJ (version 1.53c). Pollen was classified as germinated when the pollen tube exceeded the diameter of the pollen grain. The fraction of germinated pollen was calculated as the ratio of germinated to total (germinated and non-germinated) pollen grains on a 0.9 mm^2^ area.

### Fruit set, seed set, and fruit weight observations

Trusses from six different plants were harvested in each replication for measuring fruit set, seed set, and fruit mass. These plants were different from those selected for pollen quality measurements. Trusses were harvested at around 100 DAS. The number of fruits and the total weight of all fruits larger than 1 cm in diameter (weight without sepal and pedicel) were recorded for each of the trusses. Furthermore, the individual weight of the second fruit (position 2) was determined, and seeds from that fruit were counted. If the second fruit was not available, we used fruit 1 or 3.

### Statistical design and analysis

The experiment was carried out as an incomplete randomized block design (three blocks, which are three repetitions of the experiment over time) with 10 treatments. For each block, the four temperatures were randomized over five cabinets. We tested the effects of temperature and duration of the temperature stress, as well as the interaction between temperature and duration of temperature stress using a two-way unbalanced ANOVA model. The design was unbalanced because we had unequal levels of temperature stress across the durations (4, 6 or 8 days at 14°C, and 1, 3, or 4 days at 30°C and 34°C). All data were analysed in GenStat (22nd edition, 64-Bit). Mean separation was based upon Fisher’s protected LSD test. The statistical tests were all conducted using a significance level of α=0.05.

### Model development

**Figure 1:**
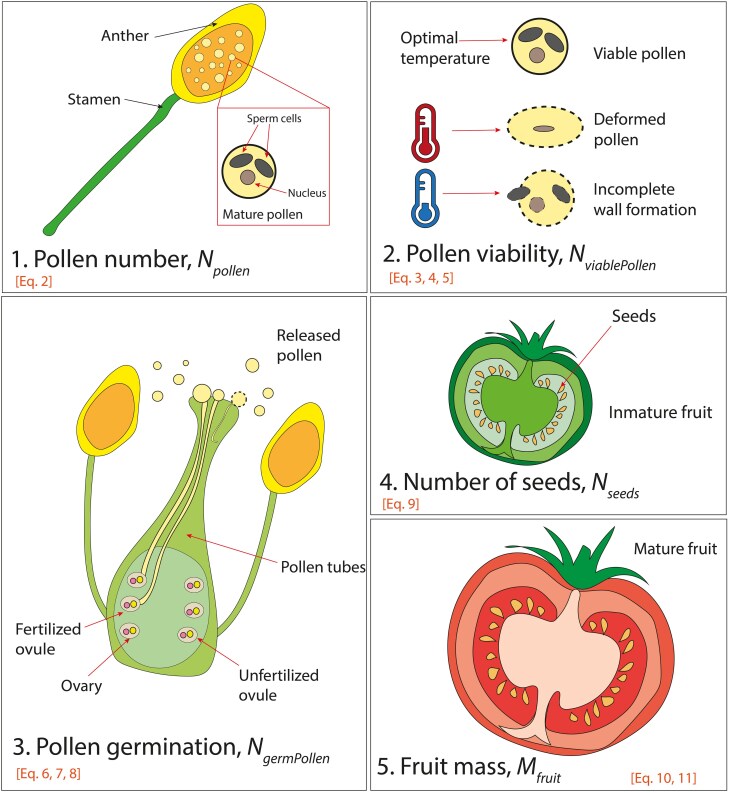
Schematic illustration of the five modelled processes (i) pollen number: pollen is developed in the anthers where microsporocytes undergo cell division until they become tricellular pollen with one nucleus and two sperm cells. (ii) Pollen viability: exposure to high or low temperatures causes abnormal pollen formation, or an incomplete wall formation leading to unviable pollen. (iii) Pollen germination: for successful fertilization mature pollen grains must land on the stigma and germinate and produce a pollen tube. High or low temperatures prevent pollen grains to germinate due to a lack of soluble sugars and starch reserves. During pollen germination, the pollen tube grows through the style until it reaches the ovule. (iv) Seed set: successful fertilization of an ovule leads to the formation of seeds (seed set). (v) Fruit growth: developing seeds in the fruit trigger auxin and gibberellin signalling pathways that promote fruit growth.

We developed a model to explain the effects of temperature and its duration on seed set and fruit mass of individual fruits. Based on plant physiology, we first identified the key processes involved in fruit formation that are influenced by temperature: (i) pollen number, (ii) pollen viability, (iii) pollen germination, (iv) seed set, and (v) fruit growth (Fig. 1). To develop the model, we conducted an experiment and measured each of these processes in plants exposed to varying temperatures and durations, then fitted a series of linear and non-linear regressions.

**Figure 2:**
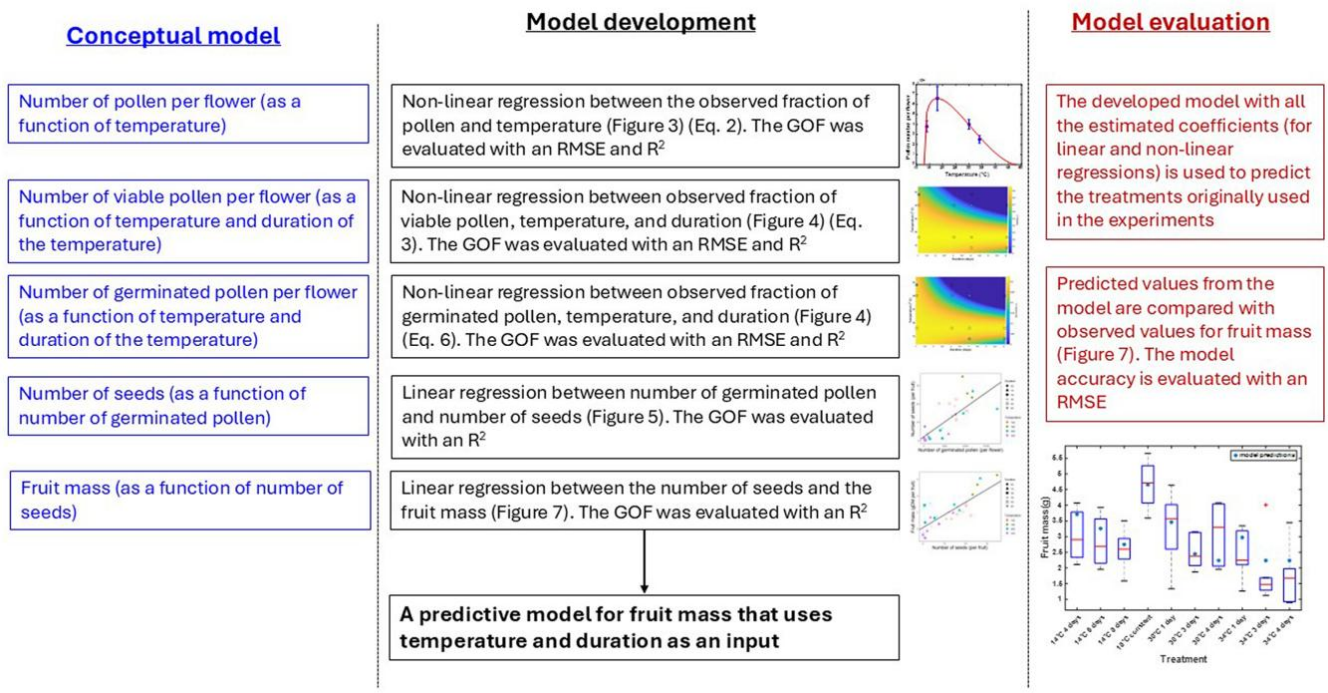
Schematic of the model development process. Based on plant physiology, key temperature-dependent processes in fruit formation were identified: (i) pollen number, (ii) pollen viability, (iii) pollen germination, (iv) seed set, and (v) fruit growth. To develop the model, we conducted an experiment measuring these processes in plants exposed to different temperatures and durations, then applied a series of linear and non-linear regressions. The final model was constructed using the derived coefficients and functions from the measured data. Model accuracy was assessed using RMSE.

Using the coefficients derived from the measured data, we constructed a model of interconnected processes: First, it predicts the effect of temperature on the total number of pollen per flower. Next, it predicts the fraction of viable pollen as a function of temperature and exposure duration. By multiplying this fraction by the total pollen count, we obtain the number of viable pollen. The model then predicts the fraction of germinated pollen under different temperature conditions, which, when multiplied by the number of viable pollen, gives the total number of germinated pollen. These values are used to predict seed number, which in turn determines fruit mass. A list of model inputs and functions is provided in Table 1, and a graphical overview of the modelled processes in Fig. 1. The complete process, from conceptualization to development and evaluation, is outlined in Fig. 2.

**Table 1:** List of functions and inputs used to model seed set and fruit mass.

Equation no.	Nomenclature	Unit	Definition
***Functions***			
2	$N_{\;pollen}(T)$	Number of pollen per flower	Number of pollen grains per flower
3	$f_{viablePollen}(T,D)$	—	Fraction of viable pollen
*4*	$p_{v}(D)$	—	Interpolation function of the duration of the temperature stress
5	$N_{viablePollen}(T,D)$	Number of viable pollen per flower	Number of viable pollen
6	$f_{germPollen}(T,D)$	—	Fraction of germinated pollen
7	$p_{g}(D)$	—	Interpolation function of the duration of the temperature stress
8	$N_{germPollen}(T,D)$	Number of germinated pollen per flower	Number of germinated pollen
9	$N_{seeds}(T,D)$	Number of seeds per fruit	Number of seeds per fruit
10, 11	$M_{\;fruit}(T,D)$	gDM per fruit	Fruit mass
***Inputs***			
	*T*	°C	Temperature
	*D*	d	Duration of the temperature stress

In the model, *N* stands for number, and *f* for fraction. For unit consistency, we differentiate with {} between viable pollen (pollen grains that are capable of fertilization) and germinated pollen (pollen grains that can grow a pollen tube).

We observed a significant effect of temperature on pollen number, with no significant interaction found between temperature and duration of a period with low or high temperature (Supplementary Table S1). The response of pollen number to temperature was asymmetrical around the optimum, where at lower temperatures, there was a more rapid decrease compared to higher temperatures. Therefore, we chose a nonsymmetric function (a beta function) as described in Yin et al. (1995) with boundary conditions (see Supplementary Text S1 for a derivation of the function). Pollen number was modelled as a function of temperature (*T*) according to


(2) $$\begin{array}{c} N_{\;pollen}(T)=e^{\mu_{np}}\cdot {\left(\frac{T-T_{b}}{T_{0}}\right)}^{\alpha_{np}}\cdot {\left(\frac{T_{c}-T}{T_{0}}\right)}^{\beta_{np}},\; \end{array}$$

with $\alpha_{np}=\beta_{np}\delta_{3}$, $\beta_{np}=\frac{\ln\;(N_{\;pollen}^{*})-\mu_{np}}{\delta_{3}\ln\;(\delta_{1})+\ln\;(\delta_{2})}$, $\delta_{1}=\frac{T_{opt}-T_{b}}{T_{0}}$, $\delta_{2}=\frac{T_{c}-T_{opt}}{T_{0}}$, and $\delta_{3}=\frac{\delta_{1}}{\delta_{2}}$. Here, $\alpha_{np}$ (−) and $\beta_{np}$ (−) are model parameters that determine the shape of the response of number of pollen to temperature, $\mu_{np}$ (−) is a model parameter that determines the magnitude of the response, $T_{b}$ (${\;}^{\circ }C$) is the base temperature, $T_{c}$ (${\;}^{\circ }C$) is the ceiling temperature, $T_{opt}$ (${\;}^{\circ }C$) is the temperature at which maximal pollen number $N_{\;pollen}^{*}$ occurs, $T_{0}$ (${\;}^{\circ }C$) is a scaling constant, set to 1 ${\;}^{\circ }C$, introduced to make temperature differences dimensionless.

Fraction of viable pollen $f_{viablePollen}$ was modelled as a function of *T* and duration (*D*)


(3) $$\begin{array}{rl} & \;f_{viablePollen}(T,D)\\ & =\max\left[0,p_{v}(D)+2(\alpha_{v}-p_{v}(D))\frac{T}{T_{opt}}-(\alpha_{v}-p_{v}(D)){\left(\frac{T}{T_{opt}}\right)}^{2}\right], \end{array}$$

where $\alpha_{v}$ (−) is the maximum viability fraction measured at $T_{opt}$ and $p_{v}(D)$ is an interpolation function of the duration of the temperature stress described by


(4) $$\begin{array}{c} \;p_{v}(D)=\;\theta_{v1}+\theta_{v2}D^{2}, \end{array}$$

where $\theta_{v1}$ (−) and $\theta_{v2}$ (days^−2^) are parameters estimated by regression (see Supplementary Text S2, Supplementary Figure S2, Supplementary Figure S3 for derivation of the functions). Here, the subscript *v* stands for viability.

Number of viable pollen ($N_{viablePollen}$, #pollen{viable} per flower) was calculated as


(5) $$N_{viablePollen}\;\;(T,D)=f_{viablePollen}N_{\;pollen}.$$

The fraction of germinated pollen ($f_{germPollen}$, −) was modelled as a function of *T* and *D* according to


(6) $$\begin{array}{rl} & \;f_{germPollen}(T,D)\\ & =max\left[0,p_{g}(D)+2(\alpha_{g}-p_{g}(D))\frac{T}{T_{opt}}-(\alpha_{g}-p_{g}(D)){\left(\frac{T}{T_{opt}}\right)}^{2}\right], \end{array}$$

where $\alpha_{g}$, (−) is the maximum germination fraction measured at optimal temperature $T_{opt}$ (°C), and $p_{g}(D)$ is an interpolation function of the duration of the temperature stress described by


(7) $$\begin{array}{c} \;p_{g}(D)=\;\theta_{g1}+\theta_{g2}D^{2}, \end{array}$$

where $\theta_{g1}$ (−) and $\theta_{g2}$ (days^−2^) are parameters estimated by regression (see Supplementary Text S3, Supplementary Figure S4 for derivation of the functions). Here, the subscript *g* stands for germination.

Number of germinated pollen ($N_{germPollen}$, #pollen{germinated} per flower) was calculated as


(8) $$\begin{array}{c} N_{germPollen}=f_{germPollen}N_{viablePollen}. \end{array}$$

The number of seeds $N_{seeds}$ (#seeds per fruit) was modelled as function of number of germinated pollen $N_{germPollen}$


(9) $$\begin{array}{c} N_{seeds}=m_{1}N_{germPollen}c_{\;flowertofruit}+b_{1}, \end{array}$$

where $m_{1}$ (#seeds per #pollen{germinated}) and $b_{1}\;$ (#seeds per fruit) are coefficients estimated from the linear regression between $N_{seeds}$ and $N_{germPollen}$. The coefficient $c_{\;flowertofruit}$ (flower per fruit) converts flowers into fruits and assumes that each flower develops into one fruit; flower abortion is not considered in this relationship but accounted for separately in a quadratic function describing the fruit set fraction as a function of temperature (see Supplementary Figure S5).

Finally, fruit mass ($M_{\;fruit}$, gDM per fruit) was modelled as a function of number of seeds ($N_{seeds})$:


(10) $$\begin{array}{c} M_{\;fruit}=m_{2}N_{seeds}+b_{2}, \end{array}$$

where $m_{2}\;$ is the slope (gDM per #seeds) and $b_{2}\;$ (gDM per fruit) is the intercept, both coefficients estimated from the linear regression between $N_{seeds}$ and $M_{\;fruit}$.

Combining Eqs. 2 to 10 results in:


(11) $$\begin{array}{c} M_{\;fruit}(T,D)=m_{2}(m_{1}N_{\;pollen}f_{viablePollen}f_{germPollen}c_{\;flowertofruit}+b_{1})+b_{2}. \end{array}$$

### Parameter estimation and model evaluation

The model consists of five main functions (Eqs. 2, 3, 6, 9, 10). The set parameters *p* that characterize each function is estimated from the experimental data using a least squares regression. For each function individually, the set of parameters found to give the best fit, denoted as $\hat{\;p}$, are estimated as the minimizer of


(12) $$\begin{array}{c} {\left(y^{obs}-y^{model}(p)\right)}^{2} \end{array}$$

where $y^{obs}$ is the observed data, and $y^{model}$ is the model predictions. A list of estimated and measured parameters is given in Table 2.

**Table 2:** List of model parameters used to simulate seed set and fruit mass.

Parameter	Value	Unit	Meaning
$\mu_{np}$	5	—	Parameter describing the magnitude of the response of the number of pollen to temperature (estimation)
$\alpha_{np}$	0.28	—	Parameter describing the shape of the response of the number of pollen to temperature (estimation)
$\beta_{np}$	1.66	—	Parameter describing the shape of the response of the number of pollen to temperature (estimation)
$N_{\;pollen}^{*}$	6.61 × 10^4^	Number of pollen per flower	Number of pollen at $T_{opt}$ (measurement)
$T_{opt}$	18	°C	Optimal temperature
$T_{c}$	48	°C	Ceiling temperature (Boote et al. 2012)
$T_{b}$	13	°C	Base temperature (Boote et al. 2012)
$T_{0}$	1	°C	Scaling constant to make temperature differences dimensionless
$\alpha_{v}$	0.94	—	Fraction of viable pollen at $T=T_{opt}$ (measurement)
$\theta_{v1}$	0.78	—	Estimated parameter used in the interpolation function of the duration of the temperature stress
$\theta_{v2}$	−0.11	d^−2^	Estimated parameter used in the interpolation function of the duration of the temperature stress
$\alpha_{g}$	0.31	—	Fraction of germinated pollen at $T=T_{opt}$ (measurement)
$\theta_{g1}$	0.34	—	Estimated parameter used in the interpolation function of the duration of the temperature stress
$\theta_{g2}$	−0.06	d^−2^	Estimated parameter used in the interpolation function of the duration of the temperature stress
$m_{1}$	1.60 × 10^−3^	Number of seeds per germinated pollen	Change in the number of seeds per unit change in the number of germinated pollen (estimation)
$b_{1}$	2.23	Number of seeds per fruit	Baseline number of seeds per fruit when there is no germinated pollen (estimation)
$c_{\;flowertofruit}$	1	Flower per fruit	Conversion factor that assumes that 1 flower is equivalent to 1 fruit
$m_{2}$	7.80 × 10^−2^	gDM per number of seeds	Change in fruit mass per unit change in number of seeds (estimation)
$b_{2}$	2.06	gDM per fruit	Baseline fruit mass when there are no seeds (estimation)

For unit consistency, we differentiate with {} between viable pollen (pollen grains that are capable of fertilization) and germinated pollen (pollen grains that can grow a pollen tube).

The goodness of fit for the entire model (Eq. 11) to the observed data was evaluated using the root mean square error (RMSE), defined as


(13) $$\begin{array}{c} RMSE=\sqrt{\frac{1}{5}\overset{5}{\underset{i=1}{\sum }}\frac{1}{10}\overset{10}{\underset{\;j=1}{\sum }}\;{\left(y_{ij}^{obs}-y_{ij}^{model}(\hat{\;p})\right)}^{2}\;}\; \end{array}$$

where *i* is and index ranging over function numbers 1 to 5 (i.e. $y_{1}=N_{\;pollen}$, $y_{2}=N_{viablePollen}$, $y_{3}=N_{germPollen}$, $y_{4}=N_{seeds}$, and $y_{5}=M_{\;fruit}$). Index *j* denotes the data point over a range of temperature and durations for each process *i* (a total of 10 treatments). $y_{ij}^{obs}$ is the observed data for function *i* at data point *j*, $y_{ij}^{model}$ is the model data for function *i* at data point *j*, and $\hat{\;p}$ is the vector of parameters previously found as the best fit.

## Results

### Pollen number

**Figure 3:**
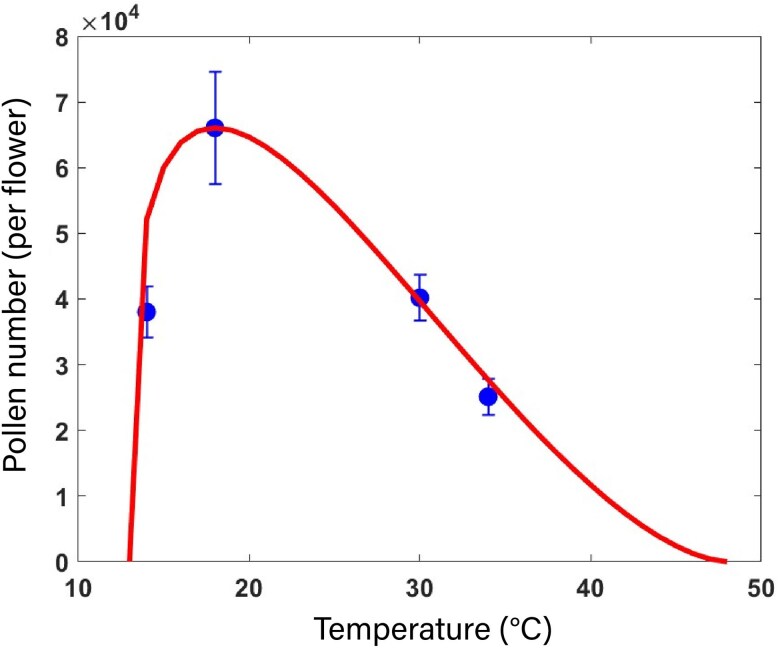
Relationship between temperature and pollen number per flower. Dots are measured data averaged over different durations (1, 3, 4, or 6 days). Data are means of 3 blocks with 4 replicate plants per block and averaged over truss 1, 2, and 3 (so each value is based on 36 plants). Error bars are ± SEM. Curve represents fitted model. The fitted model is described by Eq. 2, where $\mu_{np}=5$, $\alpha_{np}=0.28$, $\beta_{np}=1.66$, $T_{\mathrm{opt}}=18{\;}^{\circ }C$, $T_{c}=48^{\circ }C$, $T_{b}=13^{\circ }C$, and $N_{\;pollen}^{*}=6.61\times 10^{4}\;$ #pollen per flower. $RMSE=1.3\times 10^{4}$ #pollen per flower, R^2^ = 0.75.

Low and high temperatures significantly reduced the number of pollen grains produced per flower by 45%, 38%, and 30% at 14°C, 30°C, and 34°C, respectively, compared to 18°C (P<0.001, Supplementary Table S1). The asymmetrical response between pollen number and temperature showed faster declines at low temperatures (i.e. from 14°C to 18°C) than at higher temperatures. This pattern was accurately modelled using a beta function (Fig. 3, RMSE = $1.3\times 10^{4}$ #pollen per flower).

### Pollen viability

**Figure 4:**
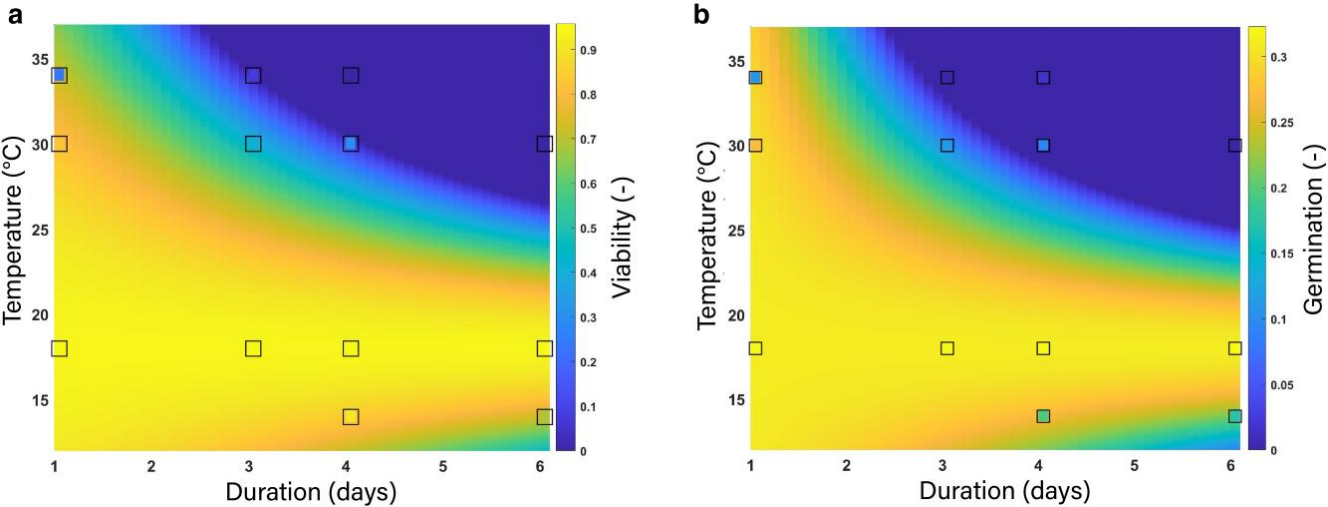
(a) Response surface of the predicted fraction of viable pollen for plants grown at different temperatures (14°C, 18°C, 30°C, or 34°C) and different durations of that temperature (0, 1, 3, 4, or 6 days) applied during the developmental phase of the flower (4 or 8 days before anthesis). Squares are measured data for 4 temperatures at different durations. The colour of the square indicates the measured viability. The model is described by Eq. 3 and Eq. 4: $f_{viablePollen}(T,D)=max\left[0,p_{v}(D)+2(\alpha_{v}-p_{v}(D))\frac{T}{T_{opt}}-(\alpha_{v}-p_{v}(D)){\left(\frac{T}{T_{opt}}\right)}^{2}\right]$, where $T_{opt}=18$°C and $p_{v}(D)=0.78-1.11D^{2}$. RMSE = 0.16 (−), R^2^ = 0.82. (b) Response surface of the predicted fraction of germinated pollen for plants grown at different temperature stress (14°C, 18°C, 30°C, 34°C) and different durations (0, 1, 3, 4 or 6 days) applied during the developmental phase of the flower (4 or 8 days before anthesis). The model is described by Eq. 6 and Eq. 7: $f_{germPollen}(T,D)=max\left[0,p_{g}(D)+2(\alpha_{g}-p_{g}(D))\frac{T}{T_{opt}}-(\alpha_{g}-p_{g}(D)){\left(\frac{T}{T_{opt}}\right)}^{2}\right]$ where $T_{opt}=18$°C, $\alpha_{g}=0.31$ (−), and $p_{g}(D)=0.34-0.06D^{2}$. RMSE = 0.06(−), R^2^ = 0.73.

Compared to 18°C constantly, pollen viability fraction was reduced by 21%, 49%, and 60% when 30°C was applied for 1, 3, and 4 days, respectively (Supplementary Table S1). At 34°C, this reduction was much more pronounced: 75%, 88%, and 98%, respectively. In contrast, a lower temperature (14°C) had only a mild effect on pollen viability. Only at longer durations (6 or 8 days) a reduction of ∼24% was observed, although not statistically significant (Supplementary Table S1). The relationship between pollen viability fraction, temperature, and duration was accurately described by Eq. 3 and Eq. 4 (Fig. 4, RMSE = 0.16 [−]).

### Pollen germination

Non-optimal temperatures reduced the fraction of pollen germination depending on the duration of the temperature stress (*P* = 0.018, Supplementary Table S1). Overall, the pollen germination fraction across treatments was low, and the maximum germination fraction observed for the control treatment (18°C) was 0.3 (Supplementary Table S1). A temperature of 30°C for 1 day did not reduce the germination fraction, but for 3 and 4 days, it reduced the germination fraction by 58% and 77%, respectively, compared to 18°C (Supplementary Table S1). After 4 days at 34°C, the germination fraction was drastically reduced by ∼99% compared to 18°C. Low temperatures had a less pronounced effect compared to high temperatures, and germination was reduced by 29%, 32%, and 52% at 4-, 6-, and 8-day durations, respectively, compared to 18°C (Supplementary Table S1). The relationship between pollen germination fraction, temperature, and duration was described accurately by Eq. 6 and Eq. 7.

### Fruit set, number of seeds and fruit mass

**Figure 5:**
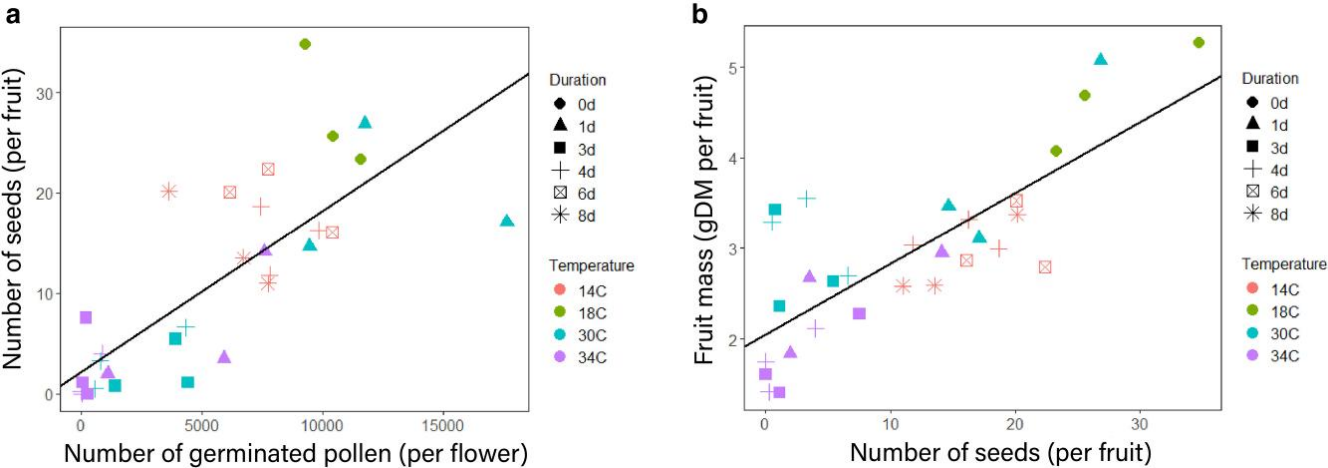
(a) Relationship between number of seeds and number of germinated pollen for 4 temperatures at different durations when applied during the development of flower buds. Symbols represent measurements; regression line: $N_{seeds}=1.60\times 10^{-3}\cdot N_{germPollen}+2.23$, $R^{2}=0.56$. Data are means of three blocks with four or six replicate plants per block. Each symbol on the graph represents data from either truss 1, truss 2, or truss 3 (so each value is based on 12 or 18 plants). (b) Relationship between fruit mass and number of seeds for 4 temperatures at different durations when applied during the development of flower buds. Symbols represent measurements; regression line: $M_{\;fruit}=7.80\times 10^{-2}\cdot N_{seeds}+2.06$, $R^{2}=0.62$. Data are means of three blocks with four or six replicate plants per block. Each symbol represents data from either truss 1, truss 2, or truss 3 (so each value is based on 12 or 18 plants).

Very high temperatures (34°C) or low temperatures (14°C) decreased the fraction of fruit set; however, temperature duration did not play a significant role (Supplementary Table S1). Fruit set was reduced by 17% and 19% at 14°C and 34°C, respectively compared to optimal temperature (Supplementary Table S2). The number of seeds was reduced by 47%, 74%, and 85% at 14°C, 30°C, and 34°C, respectively compared to 18°C (*P* < 0.001, Supplementary Table S2). Similarly, fruit mass was reduced by 38%, 36%, and 58% at 14°C, 30°C, and 34°C, respectively compared to 18°C (P<0.001, Supplementary Table S2). The number of seeds increased linearly with the increasing number of germinated pollen (*R*^2^ = 0.56, Fig. 5). Fruit mass increased linearly with an increasing number of seeds (*R*^2^ = 0.62, Fig. 5).

### Sensitive stage for pollen failure

The stage at which temperature was applied did not influence pollen production per flower. Only at a very high temperature (34°C) when temperature was applied 8 days before anthesis, pollen production was reduced by 72% compared to applying temperature 4 days before anthesis or at anthesis (Supplementary Table S3). The sensitive period for pollen viability was 4–8 days before anthesis (Supplementary Table S3), and the response was more pronounced at 30°C and 34°C compared to 14°C. Surprisingly, we did not find a specific sensitive stage for pollen germination. However, high, and low temperatures resulted in lower germination percentage compared to optimal temperatures (18°C).

### Measured vs predicted fruit mass

**Figure 6:**
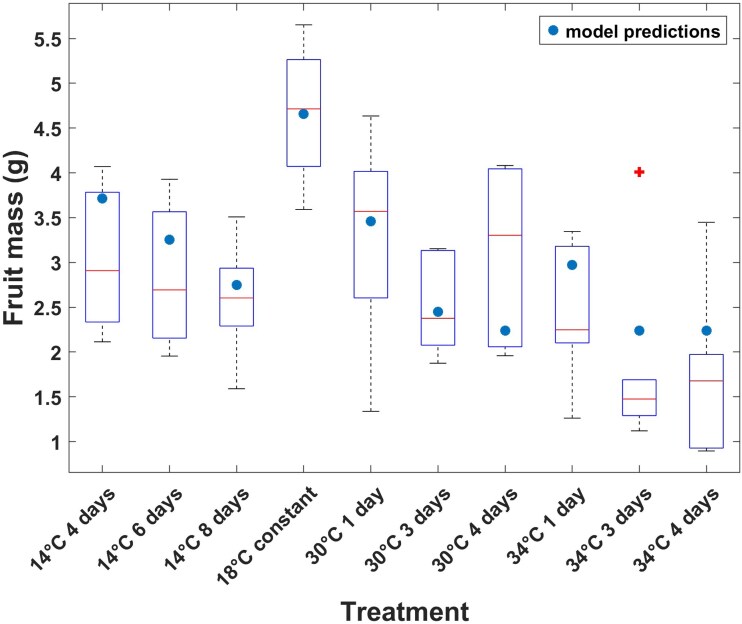
Measured (box plot) and simulated (dots) individual tomato fruit mass. Fruit mass was predicted as a function of temperature and duration when applied during the development of flower buds. According to Eq. 11. Box plots are measured data from three blocks with six replicate plants per block and averaged over truss 2 and truss 3 (so each box plot represents 36 data points). RMSE = 0.93 g. Red cross is an outlier.

Using Eq. 11, the model could accurately predict fruit fresh mass based on temperature and duration of the temperature with an overall RMSE of 0.93 g (Fig. 6). In most treatments, predictions fell within the middle 50% of the observed data, indicating a robust fit of the model to the dataset. The main deviations occurred at very high temperatures (34°C for 3–4 days), where the model slightly overestimated fruit mass. Because the model represents a cascade of linked processes, errors accumulate across steps; however, the close agreement between simulated and observed values demonstrates a strong predictive power despite the moderate *R*^2^ values for seed set–pollen germination and fruit mass–seed number relationships.

## Discussion

Flower and fruit abortion, along with seed set, are highly sensitive to temperature. These processes play a crucial role in determining yield, yet they are often not explicitly simulated in horticultural crop growth models. This omission limits the ability of current models to accurately predict growth and yield under fluctuating environmental conditions (Marcelis et al. 1998, Poorter et al. 2013). In the present study, we developed a model that links pollen development to seed set and final fruit mass, incorporating the effects of temperature on the sequential processes of pollen formation, viability, and germination.

### Pollen number is more sensitive to cold temperatures than to warm temperatures

The starting point for our model is the simulation of total number of pollen per flower. Our data show that 4°C decrease below the optimum temperature reduced pollen number as much as a 12°C increase above it, indicating greater sensitivity to cold temperatures. This likely reflects the slowing of metabolic processes and cell division at low temperatures (Ohnishi et al. 2010, Chaturvedi et al. 2021). We captured this asymmetric response using a skewed probability function (Yin et al. 1995). However, total number of pollen per flower was not correlated with seed set, fruit set, or fruit mass (Supplementary Figure S8), consistent with other observations in tomato by Sato et al. (2000) but differing from positive correlations reported in groundnut (Prasad et al. 1999) and in sweet pepper (Marcelis and Baan Hofman-Eijer 1997). This suggests that pollen quantity alone is not a reliable predictor of yield components in tomato.

### Pollen viability and germination can explain observed variation in number of seeds and fruit mass, but not in fruit set

Low and high temperature stress reduced pollen viability and germination, with greater effects at longer durations. Heat stress likely caused early tapetum degradation (Chaturvedi et al. 2021), while both heat and cold stress likely reduced pollen starch content, limiting resources for pollen germination (Pressman 2002, Firon et al. 2006). Despite the common assumption that pollen germination influences fruit set (Sato et al. 2000), our results show no such relationship (Supplementary Figure S10). Instead, germinated pollen number correlated with seed set, consistent with the significant role of seeds in fruit growth initiation via auxin or gibberellin signalling (De Jong et al. 2009). These findings indicate that pollen quality (measured by the number of viable and germinated pollen grains) can be a reliable predictor of seed set. In contrast, fruit set may occur independently of seed set (i.e. without ovule fertilization) and is more likely influenced by other environmental factors.

We observed a poor correlation between seed set and the total number of pollen per flower (Supplementary Figure S6), suggesting that heat stress reduces the probability of obtaining seeded fruits primarily by lowering pollen quality (specifically, viability and germination), rather than by decreasing the total number of pollen produced. This is logical, as non-germinating pollen cannot fertilize ovules and thus prevents seed development (Thakur et al. 2010). Hence, pollen quality is a stronger determinant of seed number than pollen quantity.

### Seed number explains fruit mass variation

Fruit mass positively correlated with seed number (Fig. 5b). The developing seeds have two functions in the early developmental stages of fruit growth: first, they favour assimilate supply to fruit (Varga and Bruinsma 1976, Bangerth and Ho 1984) and second, they trigger the fruit developmental program through the activation of auxin and gibberellin signalling pathways (Peet et al. 1997, De Jong et al. 2009). Although fruit mass positively correlates with the number of seeds at low seed numbers, this relationship can reach saturation at high seed numbers (e.g. Bakker 1989, Table 5) We did no observe saturation probably because seed numbers remained below the threshold where assimilate competition among fruits limits growth. Unlike sweet pepper (Marcelis and Baan Hofman-Eijer (1997), in our study on tomato fruit set was not correlated with seed number (Supplementary Figure S11).

### Temperature-sensitive period for fruit set and pollen development

Timing of the temperature stress only affected pollen viability (Supplementary Table S3). The greatest reductions occurred when heat was applied 4–8 days before anthesis, compared to applying it at anthesis. Neither pollen quantity nor germination showed stage-dependent temperature effects, except under high temperatures (34°C) applied 8 days before anthesis, which reduced pollen production by almost 72%. In commercial tomato cultivation, plants grown in high-wire systems have trusses at different developmental stages simultaneously. Therefore, the key challenge is to track each truss’s developmental stage precisely, in order to determine its sensitivity to temperature stress, thereby allowing accurate yield prediction and targeted climate management.

### Temperature effects on fruit set are independent of pollen quality

Fruit set followed a quadratic temperature response (Supplementary Figure S5) and showed only a weak association with seed number and fruit mass (Supplementary Figure S12). This suggests that, in our study, temperature influences fruit set independently of pollen quality, consistent with the capacity for fruit set and growth to occur without pollination (Pandolfini et al. 2009). In contrast, seed development requires successful fertilization, including the formation of viable pollen, pollen germination and tube growth, and the delivery of sperm cells (Ruan et al. 2012).

The model developed in this study incorporates sequential processes that determine final fruit mass. Fruit set was not included, as no clear correlation between fruit set and individual fruit mass was observed. However, predicting yield at the truss level still requires accounting for the fraction of fruit set, since yield reductions may result from fewer fruits, smaller fruits, or both. Additionally, the model assumes that reproductive tissue temperature equals ambient air temperature, though in reality microclimate effects can raise tissue temperature by up to 4°C (Savvides et al. 2013) increasing pollen sterility (Tian et al. 2010, Julia and Dingkuhn 2013). Elevated CO_2_ levels can reduce stomatal conductance, raising canopy temperature by 1 to 2°C, often resulting in greater sterility (Horie 1993, Yoshimoto et al. 2005).

Microclimate around the flower can affect pollination, with high humidity keeping pollen in the anthers and low humidity reducing adhesion (Picken 1984). In vitro germination assays are useful proxies but may not fully reflect *in vivo* performance, where the style and stigma influence pollen-tube growth through biochemical signals and adhesion (Mollet et al. 2000). For example, pollen tubes often grow slower *in vitro* (Johnson et al. 2019) and in petunia, style effects are absent *in vitro* but strong *in vivo* (Kato et al. 2022). Although *in vivo* germination tests better reflect natural interactions and fertilization potential, they remain technically challenging, difficult to control, and less suited for isolating specific factors.

### Implications for yield prediction

In our approach, yield at the individual-fruit level is modelled under the assumption that each flower develops into a fruit. Yield reductions under suboptimal temperatures are then attributed to fewer seeds per fruit and the resulting lower fruit mass. To account for flower abortion, we could additionally model fruit set fraction as a function of temperature (e.g. Supplementary Figure S5). By combining these two models, yield per truss can be estimated as the product of flower number per truss, fruit set fraction, and average fruit weight. This framework would allow us to disentangle the processes underlying yield reduction. For example, under heat stress (30°C) for 4 days, the predicted individual fruit mass was 2.9 g with a relatively high fruit set fraction of 0.9, resulting in approximately 23.2 gDM per truss (assuming a maximum of nine flowers per truss). Under cold stress (14°C) for the same duration, individual fruit mass was slightly higher at approximately 3.2 g, but fruit set dropped to 0.7, yielding approximately 20.1 gDM per truss. Thus, short periods of heat or cold stress can produce similar yields, but for different reasons: heat stress reduces fruit mass, whereas cold stress reduces both fruit set and fruit mass. These results highlight that relying solely on temperature effects on fruit set to predict yield can be misleading. Indirect effects of temperature, such as reduced fruit size due to a lower number of seeds per fruit, should also be considered to improve predictions accuracy. Currently, the model represents a single flower at a specific developmental stage. For application under field or greenhouse conditions with flowers at varying stages, it could be integrated into a crop-level framework tracking the developmental stage of each flower, allowing prediction of fruit set and potential fruit size for each truss.

Our model captures the sequential processes that determine fruit mass. A simpler alternative is a multiple regression with an additive structure using temperature, duration of exposure, and their interaction as predictors. This approach fits the data similarly (Supplementary Figure S14) but lacks biological interpretability, ignoring the multiplicative progression from total to viable to germinated pollen. By modelling each step explicitly, our framework includes the underlying physiological processes and identifies which processes are most temperature-sensitive or have the greatest impact on fruit mass and number of seeds, providing mechanistic insights.

## Conclusions

This work represents an important first step towards integrating reproductive processes into growth models for horticultural crops, particularly in the context of fluctuating climate. As greenhouse climate control becomes more flexible, future research should focus on identifying and incorporating the key factors and processes that influence yield predictions. A major challenge will be capturing microclimate effects, since reproductive tissue temperature can raise by several degrees above air temperature due to factors such as reduced stomatal conductance. Additionally future models could greatly be improved by integrating genotype-specific calibration data. Another important consideration is the time of day when reproductive organs are most sensitive, since certain periods might be more sensitive than others. Together, these advancements would not only improve model accuracy but also provide insights into the phenotypic plasticity and the mechanisms crops use to cope with fluctuating environmental conditions.

## Supplementary Material

plag004_Supplementary_Data

## Data Availability

Raw data, R, and Matlab code are available online at https://github.com/crisloop/Too-cold-or-too-warm-.git

## References

[plag004-B1] Alexander MP . Differential staining of aborted and non aborted pollen. Stain Technol 1969;44:117–22. 10.3109/10520296909063335 4181665

[plag004-B2] Bakker JC . The effects of air humidity on flowering, fruit set, seed set and fruit growth of glasshouse sweet pepper (*Capsicum annuum* L.). Sci Hortic 1989;40:1–8. 10.1016/0304-4238(89)90002-2

[plag004-B3] Bangerth F, Ho LC. Fruit position and fruit set sequence in a truss as factors determining final size of tomato fruits. Ann Bot 1984;53:315–20. 10.1093/oxfordjournals.aob.a086695

[plag004-B4] Bohner J, Bangerth F. Cell number, cell size and hormone levels in semi-isogenic mutants of *Lycopersicon pimpinellifolium* differing in fruit size. Physiol Plant 1988;72:316–20. 10.1111/j.1399-3054.1988.tb05839.x

[plag004-B5] Boote KJ, Jones JW, White JW et al Putting mechanisms into crop production models. Plant Cell Environ 2013;36:1658–72. 10.1111/pce.12119 23600481

[plag004-B6] Boote KJ, Rybak MR, Scholberg JMS et al Improving the CROPGRO-tomato model for predicting growth and yield response to temperature. HortScience 2012;47:1038–49. 10.21273/HORTSCI.47.8.1038

[plag004-B7] Chaturvedi P, Wiese AJ, Ghatak A et al Heat stress response mechanisms in pollen development. New Phytol 2021;231:571–85. 10.1111/nph.17380 33818773 PMC9292940

[plag004-B8] Coast O, Murdoch AJ, Ellis RH et al Resilience of rice (*Oryza* spp.) pollen germination and tube growth to temperature stress. Plant Cell Environ 2016;39:26–37. 10.1111/pce.12475 25346255

[plag004-B9] De Jong M, Mariani C, Vriezen WH. The role of auxin and gibberellin in tomato fruit set. J Exp Bot 2009;60:1523–32. 10.1093/jxb/erp094 19321650

[plag004-B10] Firon N, Shaked R, Peet MM et al Pollen grains of heat tolerant tomato cultivars retain higher carbohydrate concentration under heat stress conditions. Sci Hortic 2006;109:212–7. 10.1016/j.scienta.2006.03.007

[plag004-B11] Hedhly A . Sensitivity of flowering plant gametophytes to temperature fluctuations. Environ Exp Bot 2011;74:9–16. 10.1016/j.envexpbot.2011.03.016

[plag004-B12] Heuvelink E . Dry matter partitioning in tomato: validation of a dynamic simulation model. Ann Bot 1996;77:71–80. 10.1006/anbo.1996.0009

[plag004-B13] Horie T . Predicting the effects of climatic variation and elevated CO2 on rice yield in Japan. J Agric Meteorol 1993;48:567–74. 10.2480/agrmet.48.567

[plag004-B14] Johnson MA, Harper JF, Palanivelu R. A fruitful journey: pollen tube navigation from germination to fertilization. Annu Rev Plant Biol 2019;70:809–37. 10.1146/annurev-arplant-050718-100133 30822112

[plag004-B15] Julia C, Dingkuhn M. Predicting temperature induced sterility of rice spikelets requires simulation of crop-generated microclimate. Eur J Agron 2013;49:50–60. 10.1016/j.eja.2013.03.006

[plag004-B16] Kato M, Watanabe H, Hoshino Y. Evaluation of pollen tube growth ability in *Petunia* species having different style lengths. Plant Biotechnology 2022;39:85–92. 10.5511/plantbiotechnology.21.1113a 35937536 PMC9300434

[plag004-B17] Kearns CA, Inouye DW. Techniques for Pollination Biologists. Boulder, CO: University Press of Colorado, 1993.

[plag004-B18] Marcelis LFM, Baan Hofman-Eijer LR. Effects of seed number on competition and dominance among fruits in *Capsicum annuum* L. Ann Bot 1997;79:687–93. 10.1006/anbo.1997.0398

[plag004-B19] Marcelis LFM, Heuvelink E, Goudriaan J. Modelling biomass production and yield of horticultural crops: a review. Sci Hortic 1998;74:83–111. 10.1016/S0304-4238(98)00083-1

[plag004-B20] Mollet J-C, Park S-Y, Nothnagel EA et al A lily stylar pectin is necessary for pollen tube adhesion to an in vitro stylar matrix. Plant Cell 2000;12:1737–49. 10.1105/tpc.12.9.1737 11006344 PMC149082

[plag004-B21] Ohnishi S, Miyoshi T, Shirai S. Low temperature stress at different flower developmental stages affects pollen development, pollination, and pod set in soybean. Environ Exp Bot 2010;69:56–62. 10.1016/j.envexpbot.2010.02.007

[plag004-B22] Okello RCO, Heuvelink E, De Visser PHB et al What drives fruit growth? Funct Plant Biol 2012;42:817–27. 10.1071/FP15060 32480724

[plag004-B23] Pandolfini T, Molesini B, Spena A. Parthenocarpy in Crop Plants. Chichester: Wiley, 2009. 10.1002/9781119312994.apr0416

[plag004-B24] Peet MM, Sato S, Gardner RG. Comparing heat stress effects on male-fertile and male-sterile tomatoes. Plant Cell Environ 1998;21:225–31. 10.1046/j.1365-3040.1998.00281.x

[plag004-B25] Peet MM, Willits DH, Gardner R. Response of ovule development and post-pollen production processes in male-sterile tomatoes to chronic, sub-acute high temperature stress. J Exp Bot 1997;48:101–11. 10.1093/jxb/48.1.101

[plag004-B26] Pereira J, Revi A, Rose S et al IPCC summary for policy makers. Morgan Wairiu 2022;1:3–33. 10.1017/9781009325844.001

[plag004-B27] Pham D, Hoshikawa K, Fujita S et al A tomato heat-tolerant mutant shows improved pollen fertility and fruit-setting under long-term ambient high temperature. Environ Exp Bot 2020;178:104150. 10.1016/j.envexpbot.2020.104150

[plag004-B28] Picken AJF . A review of pollination and fruit set in the tomato (*Lycopersicon esculentum* mill.). J Hortic Sci 1984;59:1–13. 10.1080/00221589.1984.11515163

[plag004-B29] Poorter H, Anten NPR, Marcelis LFM. Physiological mechanisms in plant growth models: do we need a supra-cellular systems biology approach? Plant Cell Environ 2013;36:1673–90. 10.1111/pce.12123 23611725

[plag004-B30] Prasad V, Craufurd P, Summerfield R. Fruit number in relation to pollen production and viability in groundnut exposed to short episodes of heat stress. Ann Bot 1999;84:381–6. 10.1006/anbo.1999.0926

[plag004-B31] Pressman E . The effect of heat stress on tomato pollen characteristics is associated with changes in carbohydrate concentration in the developing anthers. Ann Bot 2002;90:631–6. 10.1093/aob/mcf240 12466104 PMC4240456

[plag004-B32] Ruan YL, Patrick JW, Bouzayen M et al Molecular regulation of seed and fruit set. Trends Plant Sci 2012;17:656–65. 10.1016/j.tplants.2012.06.005 22776090

[plag004-B33] Santiago JP, Sharkey TD. Pollen development at high temperature and role of carbon and nitrogen metabolites. Plant Cell Environ 2019;42:2759–75. 10.1111/pce.13576 31077385

[plag004-B34] Sato S, Peet MM, Thomas JF. Physiological factors limit fruit set of tomato under chronic, mild heat stress. Plant Cell Environ 2000;23:719–26. 10.1046/j.1365-3040.2000.00589.x

[plag004-B35] Sato S, Peet MM, Thomas JF. Determining critical pre- and post-anthesis periods and physiological processes in *Lycopersicon esculentum* mill. Exposed to moderately elevated temperatures. J Exp Bot 2002;53:1187–95. 10.1093/jexbot/53.371.1187 11971929

[plag004-B36] Savvides A, van Ieperen W, Dieleman JA et al Meristem temperature substantially deviates from air temperature even in moderate environments: is the magnitude of this deviation species-specific? Plant Cell Environ 2013;36:1950–60. 10.1111/pce.12101 23509944

[plag004-B37] Singh V, Nguyen CT, McLean G et al Quantifying high temperature risks and their potential effects on sorghum production in Australia. Field Crops Res 2017;211:77–88. 10.1016/j.fcr.2017.06.012

[plag004-B38] Thakur P, Kumar S, Malik JA et al Cold stress effects on reproductive development in grain crops: an overview. Environ Exp Bot 2010;67:429–43. 10.1016/j.envexpbot.2009.09.004

[plag004-B39] Tian X, Matsui T, Li S et al Heat-induced floret sterility of hybrid rice (*Oryza sativa* L.) cultivars under humid and low wind conditionsin the field of Jianghan Basin, China. Plant Prod Sci 2010;13:243–51. 10.1626/pps.13.243

[plag004-B40] Vanthoor BHE, Visser PHBD, Stanghellini C et al A methodology for model-based greenhouse design: part 2, description and validation of a tomato yield model. Biosyst Eng 2011;110:378–95. 10.1016/j.biosystemseng.2011.08.005

[plag004-B41] Varga A, Bruinsma J. Roles of seeds and auxins in tomato fruit growth. Zeitschr Pflanzenphysiol 1976;80:95–104. 10.1016/s0044-328x(76)80146-8

[plag004-B42] Yin X, Kropff MJ, McLaren G et al A nonlinear model for crop development as a function of temperature. Agric For Meteorol 1995;77:1–16. 10.1016/0168-1923(95)02236-Q

[plag004-B43] Yoshimoto M, Oue H, Takahashi N et al The effects of FACE (free-air CO_2_ enrichment) on temperatures and transpiration of rice panicles at flowering stage. J Agric Meteorol 2005;60:597–600. 10.2480/agrmet.597

[plag004-B44] Zinn KE, Tunc-ozdemir M, Harper JF. Temperature stress and plant sexual reproduction: uncovering the weakest links. J Exp Bot 2010;61:1959–68. 10.1093/jxb/erq053 20351019 PMC2917059

