# Supplementary material for: Too cold or too warm? Modelling seed set and fruit mass based on the effect of temperature on pollen quality
Source: AoB Plants. 2026 Feb 2;18(1):plag004. doi: 10.1093/aobpla/plag004 (PMC12910505; doi:10.1093/aobpla/plag004)
Supplement: plag004_Supplementary_Data [file plag004_supplementary_data.docx]

## Supporting information


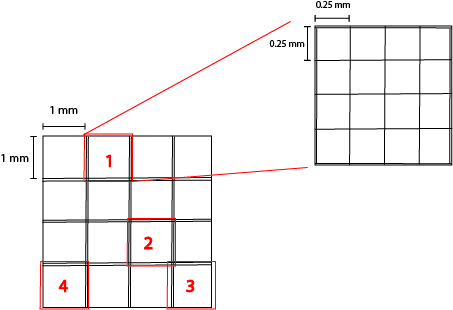


**Figure S1** Schematic representation of the Fuchs-Rosenthal haemocytometer (4 x 4 x 0.4 mm grid, 3.2 mm3). The counting chamber was divided into 16 large squares (1 x 1 mm, 1 mm^2^), and each square was further divided into16 small squares (0.25 x 0.25 mm, 0.0625 mm^2^). Two large squares (1 mm^2^) were randomly selected per load, and pictures were made, to further count the pollen with a cell counter plugin of Fiji - ImageJ (version 1.53c). For each sample (each flower), the haemocytometer was loaded two times, therefore the average pollen number per sample is based in a total of 4 pictures.

| **Table S1** Interaction effect of temperature × duration on pollen number per flower, pollen viability fraction, pollen germination fraction, fraction of fruit set, fruit weight and number of seeds per fruit. Data are means of 3 blocks with 4 or 6 replicate plants per block and averaged over truss 2 and truss 3 (so each value is based on 24 plants). | | | | | | | |
| --- | --- | --- | --- | --- | --- | --- | --- |
| Duration | Temperature (ºC) | Pollen number (#) | Viability fraction (-) | Germination fraction (-) | Fruit set fraction (-) | Fruit mass (g) | Number of seeds (#) |
|  |  |  |  |  |  |  |  |
| Constant | 18 | 63,467 | 0.95 | 0.31 d | 0.83 | 4.7 | 29 |
|  |  |  |  |  |  |  |  |
| 1 day | 30 | 34,661 | 0.75 | 0.29 d | 0.83 | 3.3 | 16 |
|  | 34 | 22,151 | 0.23 | 0.07 ab | 0.71 | 2.4 | 8.0 |
|  |  |  |  |  |  |  |  |
| 3 days | 30 | 41,922 | 0.48 | 0.13 bc | 0.85 | 2.5 | 3.2 |
|  | 34 | 26,365 | 0.11 | 0.001 a | 0.61 | 1.8 | 4.3 |
|  |  |  |  |  |  |  |  |
| 4 days | 14 | 32,484 | 0.89 | 0.22 cd | 0.70 | 3.0 | 15 |
|  | 30 | 39,474 | 0.37 | 0.07 ab | 0.83 | 3.1 | 4.9 |
|  | 34 | 15,854 | 0.009 | 0.01 a | 0.70 | 1.8 | 2.1 |
|  |  |  |  |  |  |  |  |
| 6 days | 14 | 36,344 | 0.74 | 0.21 cd | 0.68 | 2.8 | 19 |
|  |  |  |  |  |  |  |  |
| 8 days | 14 | 39,078 | 0.73 | 0.15 bc | 0.69 | 2.6 | 12 |
| F-probability interaction (temperature × duration) | | 0.596 | 0.235 | 0.018 | 0.378 | 0.609 | 0.341 |
| Standard error of the mean (SEM) | | 6,691 | 0.0521 | 0.0279 | 0.07 | 0.400 | 4.352 |

**Text S1: Derivation of the** $\boldsymbol{N}_{\boldsymbol{pollen}}$ **function**

Because we did not observe a clear effect of duration of the temperature stress on the number of pollen, we modelled only the effect of temperature on pollen number with the following beta function:

$$\begin{aligned} N_{pollen}\left( T \right)=e^{\mu_{np}}\cdot\left( \frac{T-T_{b}}{T_{0}} \right)^{\alpha_{np}}\cdot\left( \frac{T_{c}-T}{T_{0}} \right)^{\beta_{np}}, \#A1 \end{aligned}$$

where $N_{pollen}$ is the number of pollen in a flower and $T$ is temperature (°C). We have 3 boundary conditions:

1. Number of pollen is assumed to be maximal at $T_{opt}=18$ °C.
2. The maximum number of pollen in a flower is $N_{pollen}^{*}=66,056$ (determined experimentally). Therefore $N_{pollen}^{*}=N_{pollen}\left( T^{*} \right)=66,056$.
3. The number of pollen cannot be negative.

Condition 1 results in the requirement that $\frac{dN_{pollen}}{dT}\left( T_{opt} \right)=0$, whereas condition 2 is associated with the requirement that $N_{pollen}\left( T_{opt} \right)=N_{pollen}^{*}=66,056.$

$T_{opt}$ is the zero of the first-order derivative $N_{pollen}^{'}$ towards T of Eq. A1, which is

$$\begin{aligned} N_{pollen}^{'}=e^{\mu_{np}}\cdot\left( \frac{T_{opt}-T_{b}}{T_{0}} \right)^{\alpha_{np}}\cdot\left( \frac{T_{c}-T_{opt}}{T_{0}} \right)^{\beta_{np}}\left( \frac{\alpha_{np}\cdot T_{0}}{T_{opt}-T_{b}}- \frac{\beta_{np}\cdot T_{0}}{T_{c}-T_{opt}} \right)=0\#A2 \end{aligned}$$

solving gives

$$\begin{aligned} \alpha_{np}=\beta_{np}\left( \frac{T_{opt}-T_{b}}{T_{c}-T_{opt}} \right)=\beta_{np}\delta_{3}\#A3 \end{aligned}$$

Inserting Eq. A2 into Eq. A1 results in

$$\begin{aligned} N_{pollen}^{*}=e^{\mu_{np}}\cdot\left( \frac{T_{opt}-T_{b}}{T_{0}} \right)^{\beta_{np}\delta_{3}}\cdot\left( \frac{T_{c}-T_{opt}}{T_{0}} \right)^{\beta_{np}}= \\ e^{\mu_{np}}\cdot\delta_{1}^{\beta_{np}\delta_{3}}\cdot\delta_{2}^{\beta_{np}} \#A4 \end{aligned}$$

Solving for $\beta_{np}$ gives

$$\ln\left( N_{pollen}^{*} \right)=\ln e^{\mu_{np}}\cdot\delta_{1}^{\beta_{np}\delta_{3}}\cdot\delta_{2}^{\beta_{np}}=$$

$$\mu^{np}+\beta_{np}\delta_{3}\ln{(\delta}_{1})+\beta_{np}\ln\left( \delta_{2} \right)$$

$$\begin{aligned} \beta_{np}=\ln\left( \frac{N_{pollen}^{*}-\mu_{np}}{\delta_{3}\ln\left( \delta_{1} \right)+\ln\left( \delta_{2} \right)} \right)\#A5 \end{aligned}$$

**Text S2: Derivation of** $\boldsymbol{f}_{\boldsymbol{viablePollen}}$

To model viability fraction we started with a quadratic equation

$$\begin{aligned} f_{viablePollen}=p_{1}+p_{2}T+p_{3}T^{2},\#A6 \end{aligned}$$

which then was modified to satisfy three boundary conditions:

1. Fraction of viable pollen is assumed to be maximal at $T_{opt}=18$ °C
2. The maximum fraction of viable pollen is $\alpha_{v}=0.94$ (determined experimentally)
3. The fraction of viable pollen cannot be negative

Condition 1 results in the requirement that $\frac{df_{viablePollen}}{dT}\left( T_{opt} \right)=0$, whereas condition 2 is associated with the requirement that $f_{viablePollen}\left( T_{opt} \right)=\alpha_{v}=0.94.$

Derivation of Eq. A6 results in

$$\begin{aligned} p_{3}=-\frac{1}{2T_{opt}}p_{2},\#A7 \end{aligned}$$

and condition 2, combined with Eq. A7, results in

$$\begin{aligned} p_{2}=\frac{2\left( \alpha_{np}-p_{1} \right)}{T_{opt}}.\#A8 \end{aligned}$$

Inclusion of condition 3, treating $T_{opt}$ as fixed at 18°C, and denoting $p_{1}=p_{np}$, yields a model with one degree of freedom (parameter $p_{np}$ to be fitted to the data).

$$\begin{aligned} f_{viability}\left( T \right)=\max\left[ 0, p_{np}+2\left( \alpha_{np}-p_{np} \right)\frac{T}{T_{opt}}-\left( \alpha_{np}-p_{np} \right)\left( \frac{T}{T_{opt}} \right)^{2} \right].\#A9 \end{aligned}$$

Next, the model was extended to describe viability as a function of duration $(D)$ of the temperature. For this, we fitted Eq. A9 to 4 data sets separately, where each data set contained measured viability fractions under different temperatures at a specific temperature duration. Altogether this resulted in 4 curves, each associated with a different parameter value $p$ (Figure A2).

**Figure S2.** Relationship between temperature and viability of pollen grown at different durations of stress. Symbols represent measured data from flowers where stress was applied 4 or 8 days before anthesis. Model is described by the regression function $f_{viablePollen}(T)=\max\left[ 0,p_{v}+2\left( \alpha_{v}-p_{v} \right)\frac{T}{T_{opt}}-\left( \alpha_{v}-p_{v} \right)\left( \frac{T}{T_{opt}} \right)^{2} \right]$.

We fitted a quadratic expression with boundary conditions to durations (1, 3, 4, and 6) and the related estimated value $p$ at each duration (**Figure S3**). This relationship between parameter $p$ and the stress duration *D* was denoted $p_{v}(D)$. Here, $v$ stands for viability

$$\begin{aligned} p_{v}\left( D \right)=\theta_{v1}+\theta_{v2}D^{2}.\#A10 \end{aligned}$$


**Figure S3.** Parameter ($p$) estimation for pollen viability at different durations. Red dotted curve is the polynomial function: $p_{v}\left( D \right)=0.78-0.11D^{2}$. Blue dots are parameters obtained from a fitting at durations 1, 3,4 and 6 days.

Combining Eq. A9 and Eq. A10 provides the final model for pollen viability as function of temperature $T$ and duration $D$:

$$\begin{aligned} f_{viablePollen}\left( T,D \right)=\max\left[ 0,p_{v}\left( D \right)+2\left( \alpha_{v}-p_{v}\left( D \right) \right)\frac{T}{T_{opt}}-\left( \alpha_{v}-p_{v}\left( D \right) \right)\left( \frac{T}{T_{opt}} \right)^{2} \right]\#A11 \end{aligned}$$

**Text S3: Derivation of** $\boldsymbol{f}_{\boldsymbol{germPollen}}$

To model pollen germination fraction$f_{germPollen}$, we followed the same procedure as for $f_{viablePollen}$ (Eq. A6 to A11) following 3 boundary conditions:

1. Fraction of pollen germination is assumed to be maximal at $T_{opt}=18$ °C.
2. The maximum fraction of pollen germination is $\alpha_{g}=0.31$ (determined experimentally)
3. The fraction of pollen germination cannot be negative

Here the subscript $g$ stands for germination.

**Figure S4.** Relationship between temperature and germination of pollen grown at different durations of stress. Symbols represent measured data from flowers where stress was applied 4 or 8 days before anthesis. Model is described by the regression function $f_{germPollen}\left( T,D \right)=max\left[ 0,p_{g}\left( D \right)+2\left( \alpha_{g}-p_{g}\left( D \right) \right)\frac{T}{T_{opt}}-\left( \alpha_{g}-p_{g}\left( D \right) \right)\left( \frac{T}{T_{opt}} \right)^{2} \right]$


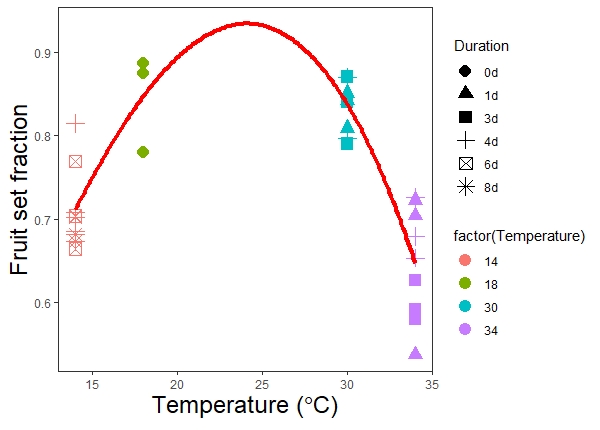


**Figure S5:** Relationship between temperature and fruit set fraction. Symbols are measured data from flowers where temperature stress was applied at anthesis and 4 and 8 days before anthesis. Data are means of 3 blocks with 4 replicate plants per block and averaged over truss 2 and truss 3 (so each value is based on 24 plants). Curve is the model prediction. Line is described by the regression function $y=-2.65\times{10}^{-3}T^{2}+1.24\times{10}^{-1}T-5.14\times{10}^{-1}$.

| **Table S2.** Temperature effect on pollen number per flower, pollen viability fraction, pollen germination fraction, fraction of fruit set, fruit weight and number of seeds per fruit. Data are means of 3 blocks with 4 or 6 replicate plants per block and averaged over truss 2 and truss 3 (so each value is based on 24 plants). | | | | | | |
| --- | --- | --- | --- | --- | --- | --- |
| Temperature (ºC) | Pollen number (#) | Viability fraction (-) | Germination fraction (-) | Fruit set fraction (-) | Fruit weight (g) | Number of seeds (#) |
| 14 | 34,575 b | 0.82 c | 0.20 | 0.69 ab | 2.90 b | 15.4 b |
| 18 | 63,467 c | 0.95 c | 0.31 | 0.83 bc | 4.67 c | 29.0 c |
| 30 | 38,798 b | 0.51 b | 0.13 | 0.83 c | 2.99 b | 7.60 a |
| 34 | 20,656 a | 0.10 a | 0.02 | 0.67 a | 1.97 a | 4.45 a |
| F-probability main effect (temperature) | <0.001 | <0.001 | <0.001 | <0.001 | <0.001 | <0.001 |
| Standard error of the mean (SEM) | 4,204 | 0.05 | 0.023 | 0.05 | 0.40 | 3.36 |

| **Table S3.** Effect of the stage at which temperature is applied (30 °C, 34 °C or 14 °C) on pollen number, viability, and germination. Data are means of 3 blocks with 4 replicate plants per block and averaged over all durations (so each value is based on 36 plants except for the control which is 12). | | | | |
| --- | --- | --- | --- | --- |
| **Pollen number (#)** | | | | |
| Period | **18 °C** | **30 °C** | **34 °C** | **14 °C** |
| Anthesis | 71,236 | 43,188 | 32,417 b | 42,139 |
| 4 days before anthesis | 62,187 | 39,767 | 33,743 b | 35,465 |
| 8 days before anthesis | 64,747 | 37,604 | 9,170 a | 36,472 |
| F-probability period | 0.93 | 0.683 | 0.007 | 0.558 |
| Standard error of the means (SEM) | 24,265 | 6,398 | 7,841 | 6576 |
| **Viability (-)** |  |  |  |  |
| Period | **18 °C** | **30 °C** | **34 °C** | **14 °C** |
| Anthesis | 0.93 | 0.80 b | 0.52 b | 0.87 b |
| 4 days before anthesis | 0.94 | 0.56 a | 0.13 a | 0.80 ab |
| 8 days before anthesis | 0.96 | 0.51 a | 0.10 a | 0.76 a |
| F-probability | 0.467 | 0.012 | 0.002 | 0.091 |
| Standard error of the mean (SEM) | 0.0204 | 0.095 | 0.1133 | 0.0510 |
| **Germination (-)** |  |  |  |  |
| Period | **18 °C** | **30 °C** | **34 °C** | **14 °C** |
| Anthesis | 0.32 | 0.20 | 0.09 | 0.19 |
| 4 days before anthesis | 0.29 | 0.16 | 0.01 | 0.21 |
| 8 days before anthesis | 0.32 | 0.14 | 0.04 | 0.17 |
| F-probability | 0.249 | 0.828 | 0.322 | 0.309 |
| Standard error of the mean (SEM) | 0.0211 | 0.0874 | 0.0544 | 0.0278 |


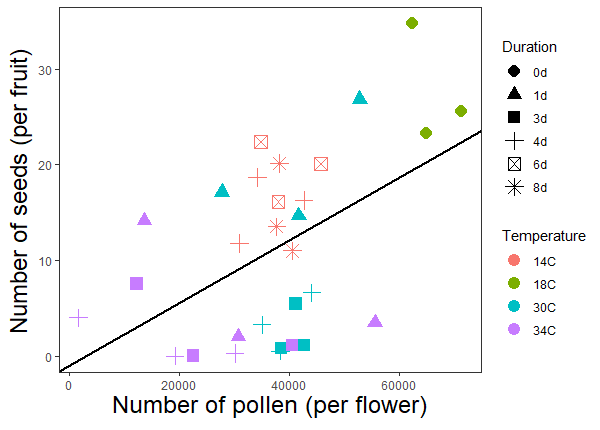


**Figure S6.** Relationship between number of seeds and total number of pollen per flower for 4 temperatures and different durations. Data are symbols and regression line: $y=3.29\times{10}^{-4}x-0.95$. $R^{2}=0.23$.


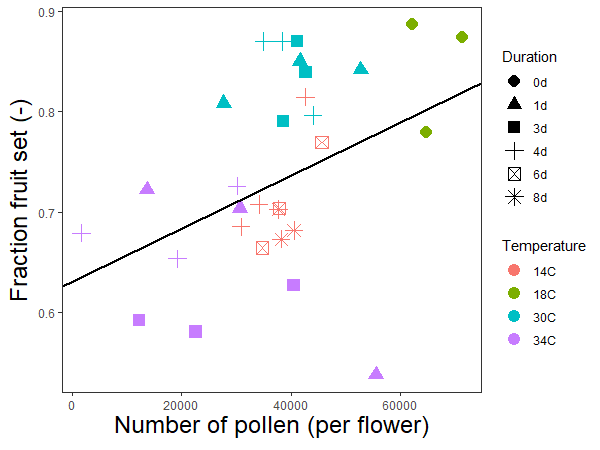


**Figure S7.** Relationship between fruit set fraction and number of pollen per flower for 4 temperatures and different durations. Data are symbols and regression line: $y=2.96\times{10}^{-6}x+0.63$. $R^{2}=0.18$.


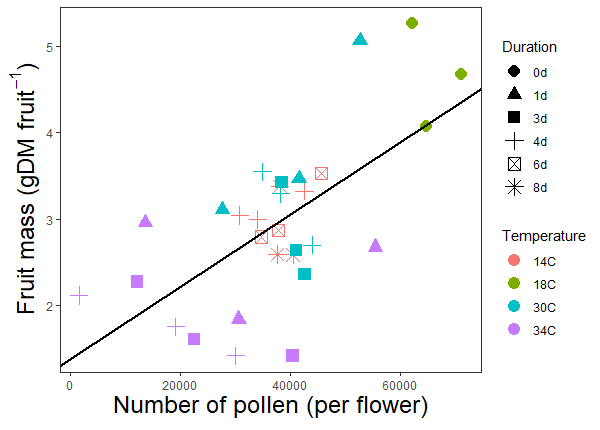


**Figure S8.** Relationship between fruit mass and total number of pollen per flower for 4 temperatures and different durations. Data are symbols and regression line: $y=4.19\times{10}^{-5}x+1.38. R^{2}=0.41$


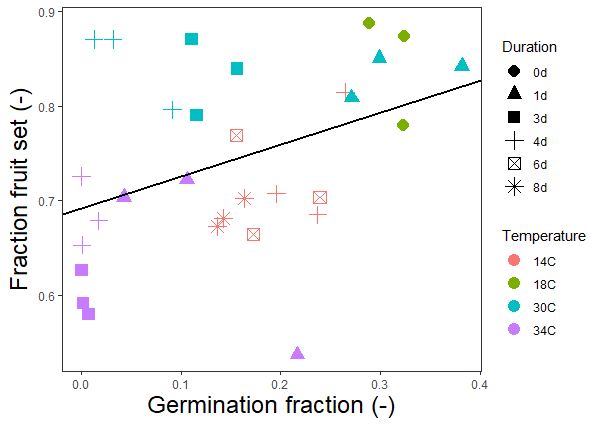


**Figure S9.** Relationship between fruit set fraction and germination fraction for 4 temperatures and different durations. Data are symbols and regression line: $y=0.34x+0.69$. $R^{2}=0.13$.


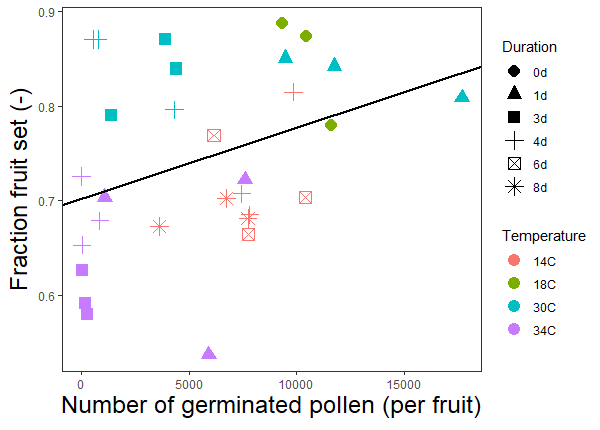


**Figure S10**. Relationship between fruit set fraction and number of germinated pollen for 4 temperatures and different durations. Data are symbols and regression line: $y=7.25\times{10}^{-6}x+7.02\times{10}^{-1}$. $R^{2}=0.07$.


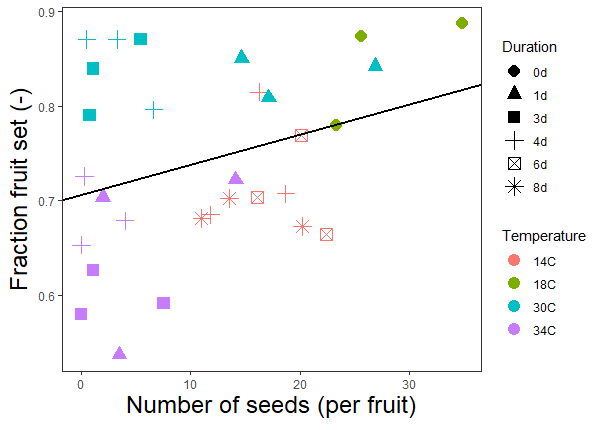


**Figure S11.** Relationship between fruit set fraction and number of seeds. Symbols represent means for 4 temperatures and different durations with regression line: $y=3.23\times{10}^{-3}x+0.7063$. $R^{2}=0.07$.


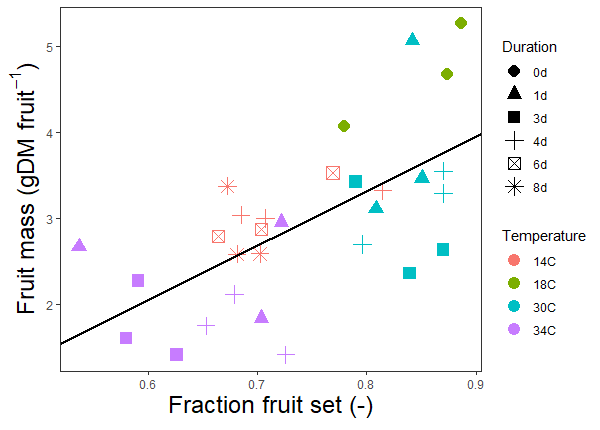


**Figure S12.** Relationship between fruit mass and fruit set fraction for 4 temperatures and different durations. Data are symbols and regression line: $y=6.346x-1.75$. $R^{2}=0.39$.


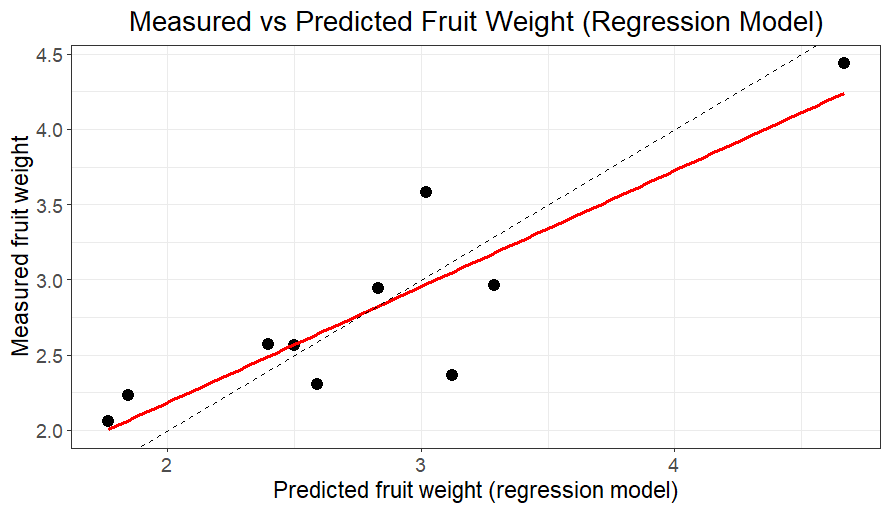


**Figure S13.** Relationship between measured and regression-predicted fruit weight based on temperature, duration, and their interaction. The solid red line shows the fitted relationship: $y=0.7701x+0.6442$, the dashed line indicates the 1:1 line. $R^{2}=0.77$.


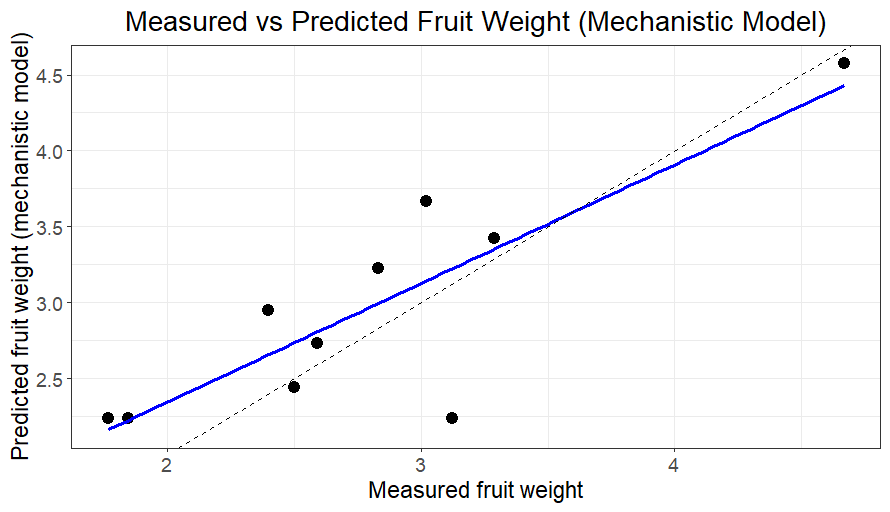


**Figure S14**. Relationship between measured weight and predicted weight using our mechanistic multiplicative model. The solid blue line shows the fitted relationship: $y=0.7813x+0.7828$, the dashed line indicates the 1:1 line. $R^{2}=0.71$.
